# Trends in mortality from Alzheimer’s disease and related dementias with hyperlipidemia in the United States from 1999 to 2020—a CDC WONDER database study

**DOI:** 10.3389/fneur.2025.1705607

**Published:** 2025-12-17

**Authors:** Junwen Wang, Kaide Xia, Dianmei Yang, Jing Wu, Longfei Liu, Ying Huang, Qing Shan, Haiwang Zhang, Yiming Wang

**Affiliations:** 1 School of Clinical Medicine, Guizhou Medical University, Guiyang, China; 2 Department of Psychosomatic Medicine, The Second People’s Hospital of Guiyang, Guiyang, China; 3 Guiyang Maternal and Child Health Care Hospital, Guiyang Children’s Hospital, Guiyang, China; 4 Department of Endocrinology and Metabolism, Affiliated Hospital of Guizhou Medical University, Guiyang, China; 5 Department of Psychiatry, Affiliated Hospital of Guizhou Medical University, Guiyang, China; 6 Department of Neurosurgery, Guizhou Provincial People’s Hospital, Guiyang, China

**Keywords:** Alzheimer’s disease and related dementias, hyperlipidemia, ASMR, CDC WONDER, AAPC

## Abstract

**Background:**

The co-occurrence of Alzheimer’s disease and related dementias (ADRD) with hyperlipidemia represents a growing public health burden amid population aging. Although both conditions have been independently linked to increased morbidity and mortality, national trends in ADRD-related mortality involving hyperlipidemia remain poorly characterized.

**Methods:**

We conducted a retrospective, population-based study using mortality data from the U.S. Centers for Disease Control and Prevention Wide-ranging Online Data for Epidemiologic Research (CDC WONDER) from 1999 to 2020. Deaths with co-listed International Classification of Diseases, Tenth Revision codes for ADRD and hyperlipidemia were identified. Age-standardized mortality rates (ASMR) were calculated per 100,000 persons using the 2000 U.S. standard population. Joinpoint regression was employed to estimate annual percentage change (APC) and average annual percentage change (AAPC) with 95% confidence intervals (CI).

**Results:**

Between 1999 and 2020, the number of deaths related to ADRD with hyperlipidemia increased from 519 to 21,969, with the ASMR rising from 0.19 to 5.32 per 100,000 (AAPC: 15.25%; 95% CI: 14.37–17.31). A sharp rise in mortality was observed after 2018 across nearly all subgroups. Males had a steeper increase than females (AAPC: 16.31% vs. 14.97%). Non-Hispanic Black individuals had the highest ASMR in 2020 (5.53 per 100,000), while Asian/Pacific Islanders had the most rapid increase (AAPC: 21.80%). Regionally, the South showed the highest burden, while the Northeast exhibited the fastest growth (AAPC: 17.77%). Rural areas had a higher ASMR than metropolitan areas (6.29 vs. 5.09 per 100,000), with comparable upward trends. Notably, individuals aged ≥85 years accounted for over half of all deaths by 2020 and exhibited the highest age-specific mortality rates.

**Conclusion:**

ADRD-related mortality involving hyperlipidemia has significantly risen in the U.S. over two decades, with notable disparities across demographics and geography, underscoring the need for public-health relevance and coordination to be evaluated in future analytic studies targeting cardiometabolic and cognitive health in high-risk populations.

## 1 Introduction

With the accelerating global aging population, the health burden of neurodegenerative and metabolic disorders has become increasingly prominent (1, 2). By 2050, deaths attributable to Alzheimer’s disease and related dementias (ADRD) in the United States are projected to reach 1.6 million, accounting for 43% of all elderly deaths—a significant increase from 600,000 cases (32%) in 2010 (3). Concurrently, hyperlipidemia, a modifiable cardiometabolic risk factor, demonstrates persistently rising prevalence (4). Epidemiological, pathological, and clinical overlaps between ADRD and hyperlipidemia suggest potential synergistic effects or comorbidity (5, 6).

Recent studies indicate that dyslipidemia may promote ADRD pathogenesis through multiple mechanisms, including β-amyloid (Aβ) deposition, aberrant tau phosphorylation, and blood–brain barrier dysfunction (7–9). Conversely, AD-associated neurodegeneration may disrupt central nervous system lipid regulation, creating a potentially interrelated process described in prior literature between metabolic disorders and cognitive impairment (10). Shared genetic susceptibility factors such as apolipoprotein E (APOE) ε4 further contribute to both conditions (11, 12).

Despite growing understanding of AD-hyperlipidemia interactions, population-level studies—particularly regarding mortality burden—remain limited. AD-hyperlipidemia comorbidity is associated with poorer health outcomes, increased healthcare expenditures, and greater caregiving demands, disproportionately affecting elderly and socioeconomically disadvantaged populations (10, 13). Existing research primarily focuses on singular disease burdens of AD or cardiovascular diseases, lacking systematic analyses of comorbid mortality trends across sex, race, urban–rural residence, and geographic regions (14).

This study examines spatiotemporal trends in age-standardized mortality rates (ASMR) and average annual percentage changes (AAPC) for ADRD-hyperlipidemia-related deaths in the U.S. (1999–2020). Using the U.S. Centers for Disease Control and Prevention’s Wide-ranging Online Data for Epidemiologic Research (CDC WONDER) database, we evaluated demographic and geographic disparities. Our findings provide critical epidemiological evidence to guide clinical strategies and region-specific public health interventions for reducing comorbid burden. Using multiple-cause-of-death data, this study descriptively reports age-standardized mortality rates for ADRD with hyperlipidemia and is hypothesis-generating rather than causal.

## 2 Methods

### 2.1 Study design and population

This study constitutes a retrospective descriptive analysis utilizing nationwide death certificate data to systematically evaluate temporal trends in mortality related to comorbid with hyperlipidemia in the United States from 1999 to 2020. This study utilized the Multiple Cause of Death Public Use Data from the CDC WONDER database,^1^ which includes legally certified death certificate records from all 50 U.S. states and the District of Columbia. The dataset provides information on contributing causes of death, along with demographic variables such as age, sex, race, urban–rural classification, and geographic region (15). This dataset has been previously validated for national mortality trend analyses involving AD in association with type 2 diabetes and pneumonia (16, 17). U.S. mortality has been coded under ICD-10 continuously since 1999. Accordingly, we used multiple-cause-of-death fields in CDC WONDER to identify ADRD (ICD-10 codes F01, F03, G30, G31) and hyperlipidemia (E78), and computed age-standardized mortality rates by direct standardization to the 2000 U.S. standard population; note that death-certificate coding does not capture lipid fractions, so LDL vs. HDL cannot be distinguished. Individuals with both ADRD and hyperlipidemia documented on death certificates were included. As this study utilized publicly available, de-identified federal data, institutional review board approval was waived, and reporting adhered to the Strengthening the Reporting of Observational Studies in Epidemiology (STROBE) guidelines.

### 2.2 Data abstraction

Mortality data were stratified by key demographic and geographic variables, including sex, race/ethnicity, urbanization level, U.S. census region, and state of residence. Race and ethnicity were categorized as American Indian or Alaska Native (AI/AN), Asian or Pacific Islander (API), Black or African American (Black), and White. Urban–rural classification followed the National Center for Health Statistics (NCHS) scheme, which defines metropolitan areas as large metro counties (population ≥1million) and medium/small metro counties (population 50,000–999,999), while nonmetropolitan (rural) areas include counties with populations under 50,000, based on 2013 U.S. Census data. Geographic regions were defined according to the U.S. Census Bureau’s classification: Northeast, Midwest, South, and West.

### 2.3 Statistical analysis

Age-standardized mortality rates (ASMR) per 100,000 population were calculated using the 2000 U.S. standard population to evaluate national trends in ADRD-with-hyperlipidemia-related deaths (18). To contextualize these deaths within the overall ADRD mortality burden, we additionally obtained, for each calendar year, the total number of deaths mentioning ADRD (using the same ICD-10 definitions as above) and calculated the proportion of these ADRD deaths that also listed hyperlipidemia (Supplementary Figure 1). Temporal trends were analyzed via the Joinpoint Regression Program (Version 5.4.0.0; National Cancer Institute, Bethesda, MD) (19). Monte Carlo permutation tests determined annual percent changes (APC) and average annual percent changes (AAPCs) with 95% confidence intervals (CI) for each AAMR segment. Trends were classified as increasing or decreasing based on deviation from the null hypothesis (no change), with statistical significance set at *p* < 0.05.

## 3 Results

### 3.1 Trends in ADRD-related mortality with hyperlipidemia

From 1999 to 2020, overall ADRD mortality showed an upward trend, but with a much more gradual increase than that observed for ADRD with hyperlipidemia. During the same period, the number of deaths attributed to ADRD with concomitant hyperlipidemia in the United States increased from 519 to 21,969. The ASMR rose from 0.19 to 5.32 per 100,000 population, with an AAPC of 15.25% (95% CI: 14.37–17.31) (Table 1). Joinpoint regression analysis identified three significant inflection points: the ASMR increased most rapidly during 1999–2005 (APC: 29.11%, *p* < 0.05), followed by decelerations to 14.20% during 2005–2011 and 3.77% during 2011–2018. However, the ASMR exhibited a renewed surge during 2018–2020 (APC: 21.65%, *p* < 0.05), indicating a substantial escalation in mortality burden in recent years (Supplementary Figure 2). To contextualize these deaths within the overall ADRD mortality burden, we additionally analyzed trends in total ADRD mortality using the same CDC WONDER dataset, stratified by sex, race, census region, and urbanization level (Supplementary Figures 4–6). While both groups exhibited a synchronized surge in mortality rates after 2018 across most subgroups, their long-term trajectories differed significantly. The total ADRD mortality showed a relatively moderate increase compared to the steep exponential rise observed in the ADRD-with-hyperlipidemia group (e.g., AAPC for total ADRD was significantly lower than the 15.25% observed in the comorbid group). This suggests that while recent external factors may have impacted both categories similarly, the specific burden of hyperlipidemia-associated ADRD has disproportionately intensified over the past two decades.

**Table 1:** Deaths and ASMRs with ADRD and hyperlipidemia by sex, race, region, and urbanization (1999–2020).

Characteristics	1999		2020		AAPC (95CI)
	Number	ASMR	Number	ASMR	
Total	519	0.19 (0.18, 0.21)	21,969	5.32 (5.25, 5.39)	15.25 (14.37–17.31)
Sex					
Female	348	0.20 (0.18, 0.22)	13,568	5.42 (5.33, 5.52)	14.97 (14.17–16.53)
Male	171	0.17 (0.15, 0.20)	8,401	5.11 (5.00, 5.22)	16.31 (14.67–19.94)
Race					
AI/AN	20^a^	1.32 (0.80, 2.07)	90	3.04 (2.44, 3.74)	5.47 (1.22–11.38)
API	22^b^	0.44 (0.28, 0.67)	735	3.58 (3.32, 3.84)	11.56 (9.65–15.29)
Black	33	0.16 (0.11, 0.22)	2024	5.53 (5.28, 5.77)	15.38 (14.21–17.59)
White	473	0.19 (0.18, 0.21)	19,120	5.42 (5.35, 5.50)	15.32 (14.46–17.31)
Census region					
Northeast	72	0.12 (0.09, 0.15)	4,091	5.13 (4.98, 5.29)	17.77 (16.18–21.85)
Midwest	157	0.24 (0.20, 0.28)	4,727	5.29 (5.13, 5.44)	14.25 (13.43–15.99)
South	197	0.21 (0.18, 0.24)	8,339	5.48 (5.36, 5.60)	15.82 (14.53–18.4)
West	93	0.18 (0.14, 0.22)	4,812	5.25 (5.10, 5.40)	14.02 (13.19–15.94)
Urbanization					
Metropolitan	410	0.18 (0.17, 0.20)	17,664	5.09 (5.02, 5.17)	15.48 (14.53–17.41)
Nonmetropolitan	109	0.20 (0.17, 0.24)	4,305	6.29 (6.10, 6.48)	15.28 (14.52–16.47)

a Data for 2008.

b Data for 2000.

Data before these years was unavailable or unreliable. ASMR, age-standardized mortality rate; ADRD, Alzheimer’s disease and related dementias; AAPC, average annual percent change.

### 3.2 Sex-stratified trends in ADRD-related mortality with hyperlipidemia

**Figure 1:**
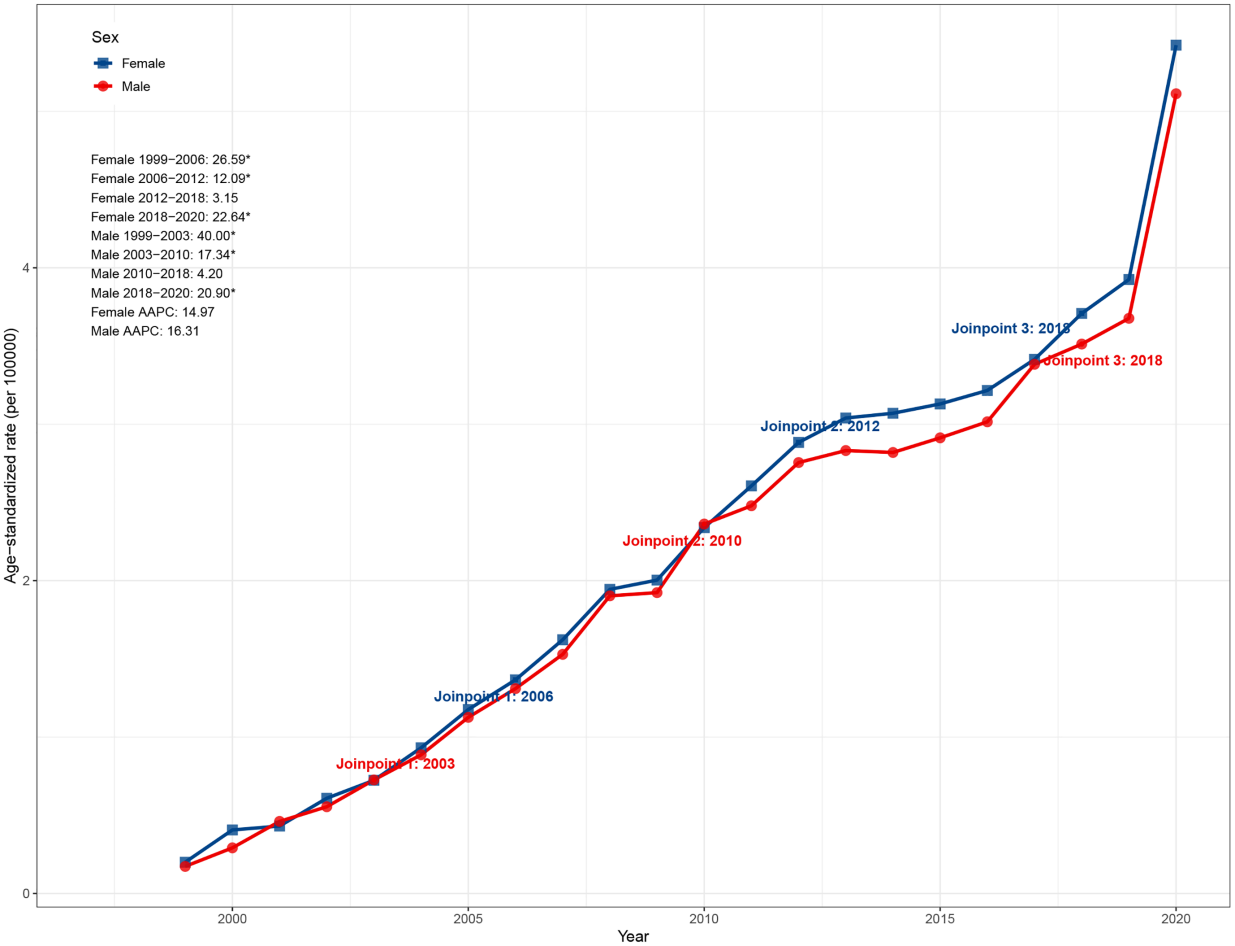
Sex-specific trends in ASMR due to ADRD and hyperlipidemia in the United States, 1999–2020. ASMR, age-standardized mortality rate; ADRD, Alzheimer’s disease and related dementias.

Sex-stratified analysis revealed that although the ASMR in females was slightly higher than in males (5.42 vs. 5.11 per 100,000 in 2020), the upward trend was more pronounced in males, with an AAPC of 16.31% (95% CI: 14.67–19.94) compared to 14.97% (95% CI: 14.17–16.53) in females. As shown in Figure 1, the ASMR in females increased most sharply during 1999–2006 (APC: 26.59%, *p* < 0.05), followed by declines to 12.09% (2006–2012) and 3.15% (2012–2018), before rebounding during 2018–2020 (22.64%, *p* < 0.05). In contrast, males exhibited a steeper initial rise during 1999–2003 (APC: 40.00%, *p* < 0.05), which subsequently moderated to 17.34% (2003–2010) and 4.20% (2010–2018), followed by a significant resurgence during 2018–2020 (APC: 20.90%, *p* < 0.05) (Figure 1).

### 3.3 Race-stratified trends in ADRD-related mortality with hyperlipidemia

**Figure 2:**
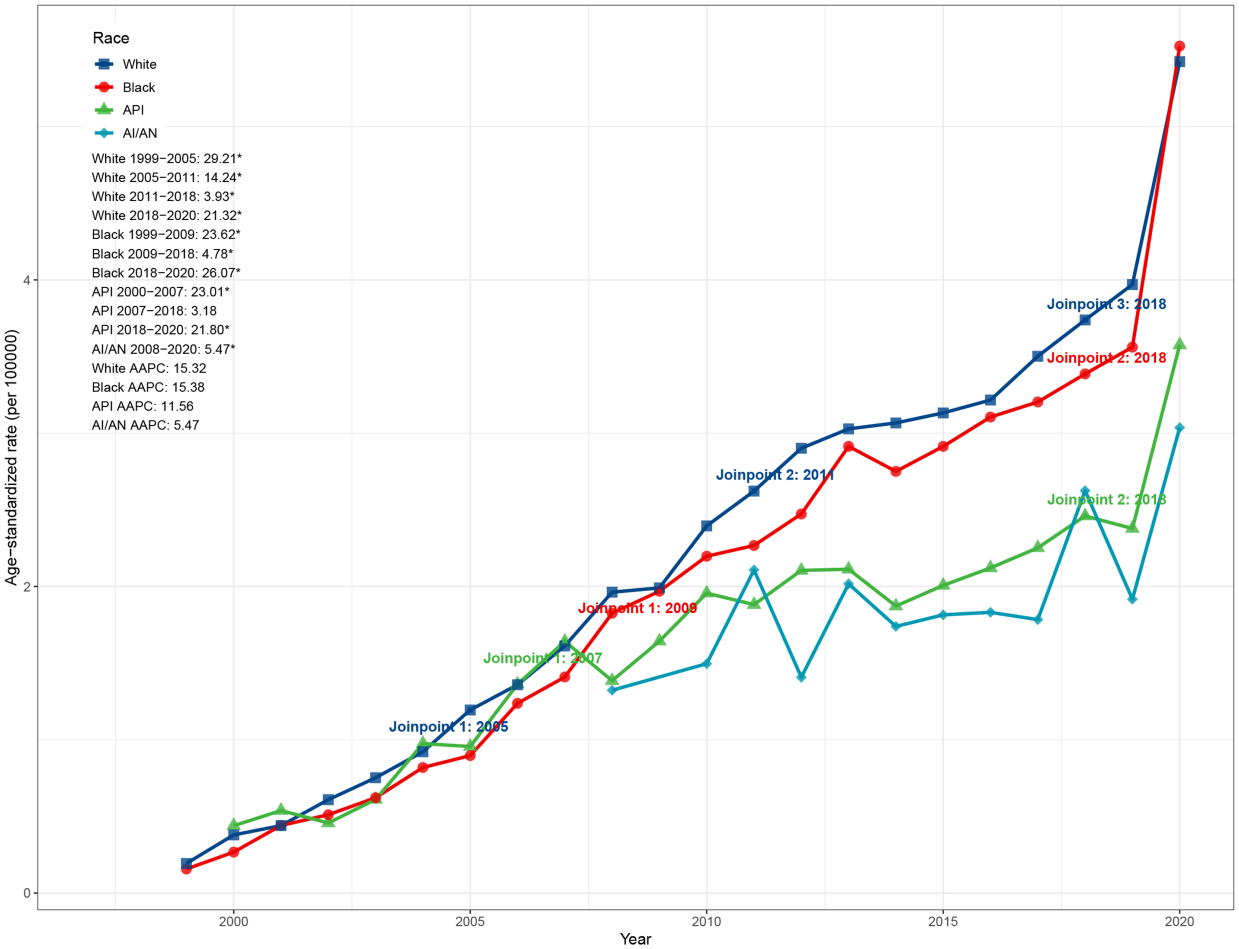
Race-specific trends in ASMR due to ADRD and hyperlipidemia in the United States, 1999–2020. ASMR, age-standardized mortality rate; ADRD, Alzheimer’s disease and related dementias.

Race-stratified analysis demonstrated that the AI/AN population had the smallest increase in ASMR, rising from 1.32 per 100,000 in 2008 (95% CI: 0.80–2.07) to 3.04 per 100,000 in 2020, with an AAPC of 5.47% (95% CI: 1.22–11.38). In 2020, the Black population exhibited the highest ASMR (5.53 per 100,000, 95% CI: 5.28–5.77) and the largest AAPC (15.38, 95% CI: 14.21–17.59). The API and White populations had ASMRs of 3.58 and 5.42 per 100,000 in 2020, with corresponding AAPCs of 11.56 and 15.32%, respectively. As illustrated in Figure 2, the ASMR in White and Black populations exhibited sustained increases since the early 2000s. The White population experienced the most rapid growth during 1999–2005 (APC: 29.21%, *p* < 0.05), followed by a marked deceleration during 2011–2018 (3.93%), before accelerating again during 2018–2020 (21.32%, *p* < 0.05). The Black population displayed a slightly delayed trend but reached a 26.07% increase during 2018–2020. In contrast, the API population showed rapid growth during 2000–2007 (APC: 23.01%, *p* < 0.05), followed by a notable slowdown (3.18%), before rebounding during 2018–2020 (21.80%, *p* < 0.05). The AI/AN population exhibited considerable fluctuations post-2008, with overall slow growth and no statistically significant inflection points (Figure 2).

### 3.4 Census region-stratified trends in ADRD-related mortality with hyperlipidemia

**Figure 3:**
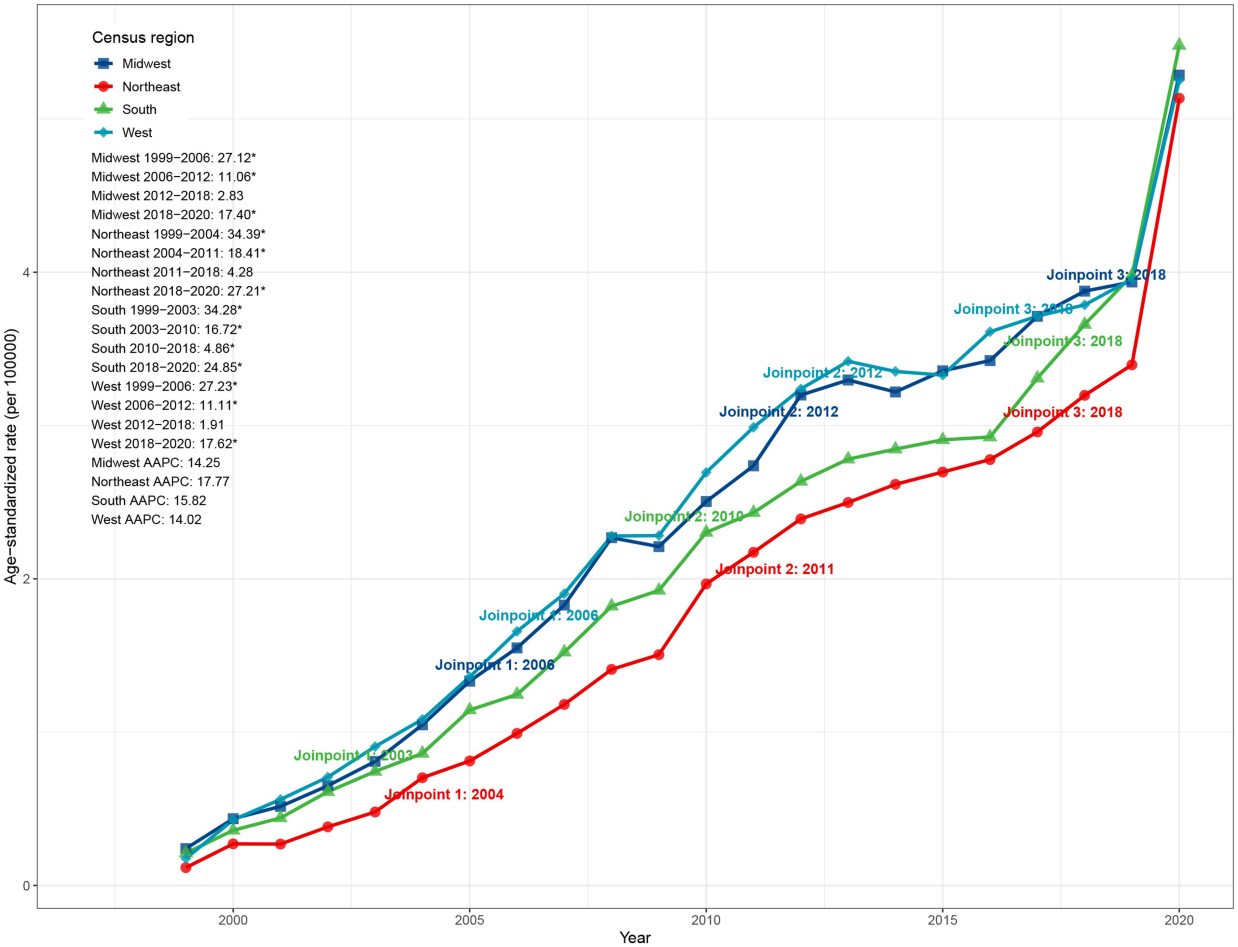
Census region trends in ASMR due to ADRD and hyperlipidemia in the United States, 1999–2020. ASMR, age-standardized mortality rate; ADRD, Alzheimer’s disease and related dementias.

When stratified by U.S. Census region, the South had the highest ASMR in 2020 (5.48 per 100,000, 95% CI: 5.36–5.60), whereas the Northeast, despite having the lowest baseline ASMR in 1999 (0.12 per 100,000, 95% CI: 0.09–0.15), exhibited the fastest growth (AAPC: 17.77, 95% CI: 16.18–21.85). As shown in Figure 3, the Northeast experienced a sharp rise in ASMR during 1999–2004 (APC: 34.39%, *p* < 0.05), followed by decelerations to 18.41% (2004–2011) and 4.28% (2011–2018), before reaccelerating during 2018–2020 (27.21%, *p* < 0.05). The South also showed a significant increase during 2003–2010 (APC: 16.72%), followed by slower growth (4.86%) during 2010–2018 and a resurgence during 2018–2020 (24.85%). The Midwest and West exhibited similar patterns, with the Midwest displaying rapid growth during 1999–2006 (27.12%) before gradual deceleration, and the West showing an increase during 2006–2012 (11.11%), a slowdown during 2012–2018 (1.91%), and renewed growth during 2018–2020 (17.62%) (Figure 3).

### 3.5 Urban–rural stratified trends in ADRD-related mortality with hyperlipidemia

**Figure 4:**
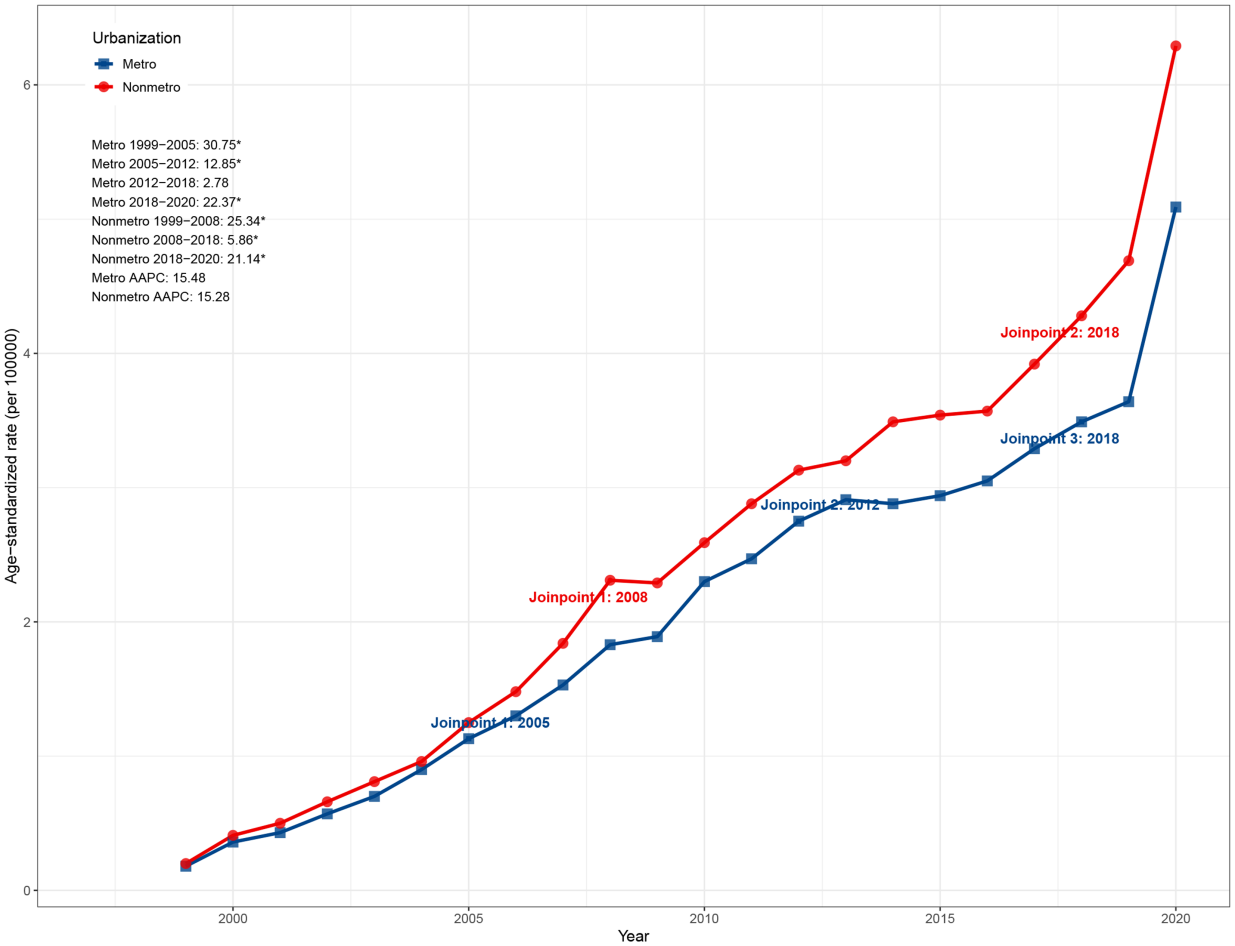
Urban–rural disparities in ASMR due to ADRD and hyperlipidemia in the United States, 1999–2020. ASMR, age-standardized mortality rate; ADRD, Alzheimer’s disease and related dementias.

In 2020, the ASMR in nonmetropolitan areas (6.29 per 100,000) exceeded that in metropolitan areas (5.09 per 100,000). Despite this disparity, the overall upward trends were comparable, with AAPCs of 15.28% (95% CI: 14.52–16.47) for nonmetropolitan areas and 15.48% (95% CI: 14.53–17.41) for metropolitan areas. As depicted in Figure 4, metropolitan areas exhibited rapid ASMR growth during 1999–2005 (APC: 30.75%, *p* < 0.05), followed by slower increases of 12.85% (2005–2012) and 2.78% (2012–2018), before rebounding during 2018–2020 (22.37%, *p* < 0.05). Nonmetropolitan areas displayed a more pronounced initial rise during 1999–2008 (APC: 35.34%, *p* < 0.05), followed by slower growth (5.86%) during 2008–2018 and a resurgence during 2018–2020 (21.14%, *p* < 0.05). Collectively, these trends indicate that while nonmetropolitan areas maintained slightly higher absolute mortality rates, both regions experienced a mid-2010s deceleration followed by a short-term rebound (Figure 4).

### 3.6 Age-stratified trends in ADRD-related mortality with hyperlipidemia

**Figure 5:**
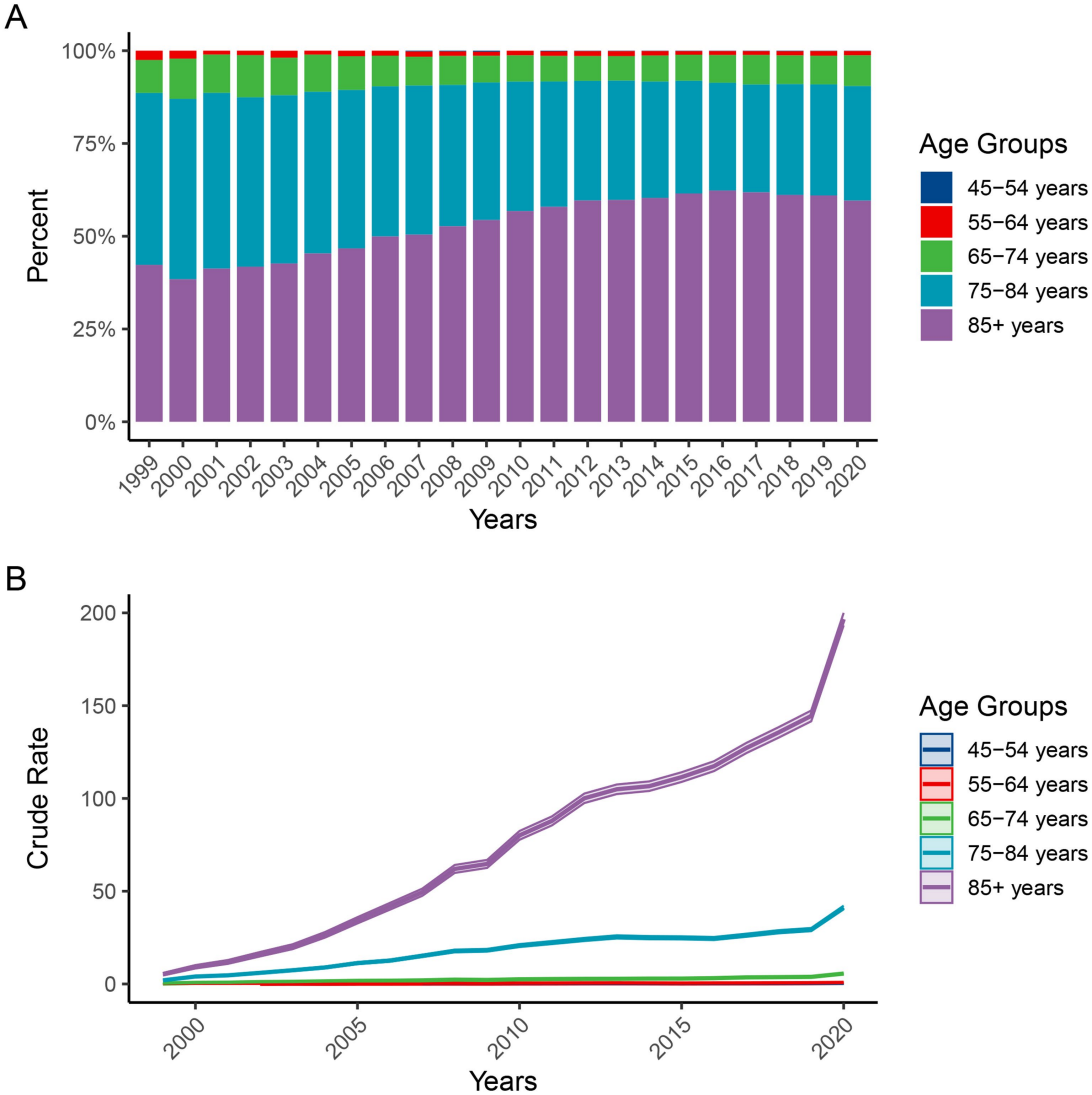
Trends in the proportion **(A)** and crude mortality rate **(B)** of ADRD-related deaths with hyperlipidemia across age groups in the United States, 1999–2020. ADRD, Alzheimer’s disease and related dementias.

Figure 5A demonstrates that individuals aged ≥85 years consistently accounted for the majority of deaths, increasing from approximately 45% in 1999 to nearly 60% in 2020. Those aged 75–84 and 65–74 years followed, whereas the proportion among individuals aged 45–64 years remained small and stable. Figure 5B further reveals that the crude mortality rate in the ≥85 age group was substantially higher than in other groups, exceeding 200 per 100,000 by 2020. The 75–84 age group also exhibited an upward trend, albeit less pronounced, whereas the remaining age groups (particularly 45–64 years) maintained low and slowly increasing rates. These findings underscore that ADRD-related mortality with hyperlipidemia predominantly affects the oldest-old population (≥85 years), which warrants prioritized public health interventions and resource allocation (Figure 5).

### 3.7 Geographic variations in ADRD-related mortality with hyperlipidemia

**Figure 6:**
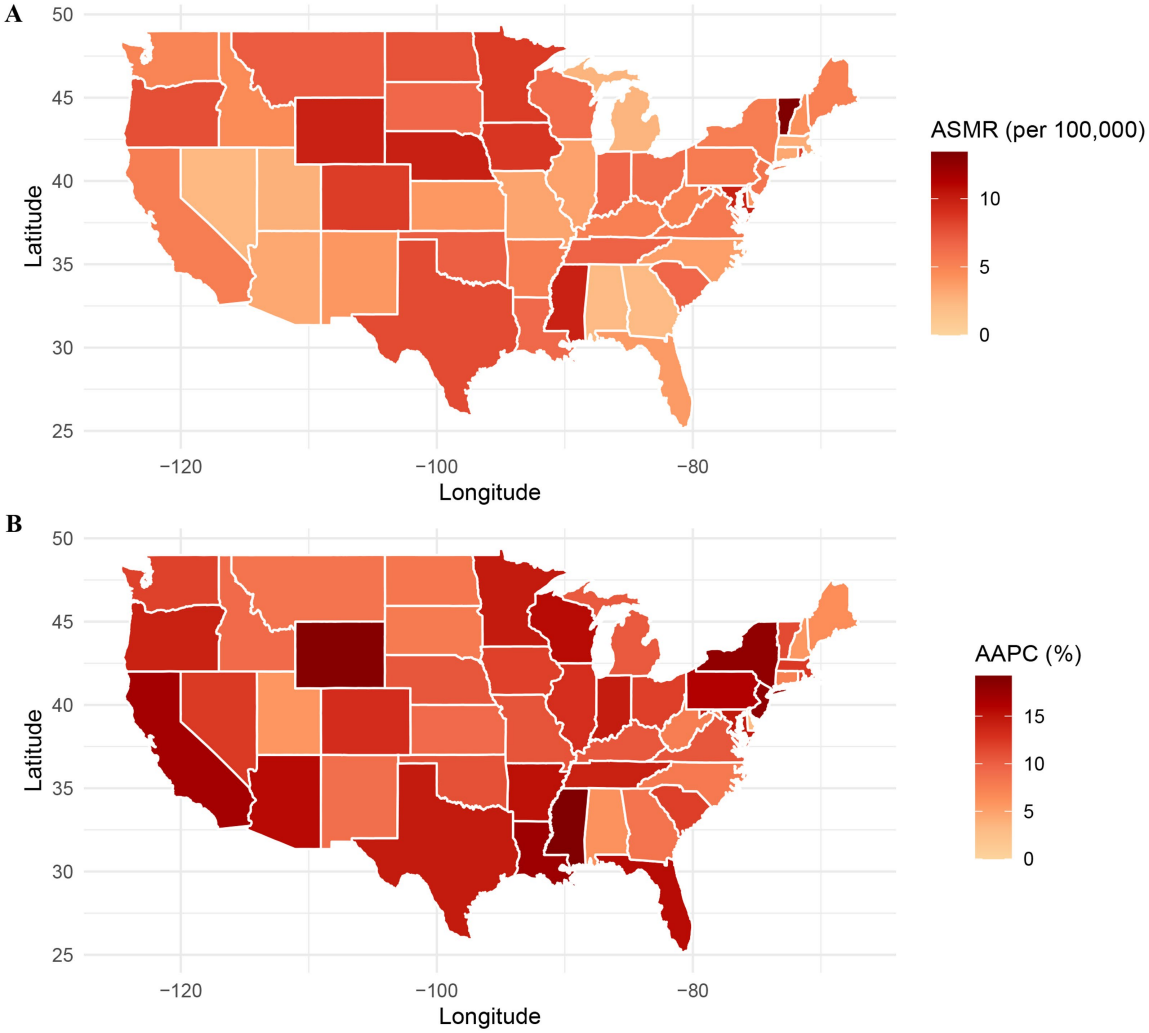
Geographic region–specific trends in ASMR **(A)** and AAPC **(B)** due to ADRD and hyperlipidemia in the United States, 1999–2020. ASMR, age-standardized mortality rate; ADRD, Alzheimer’s disease and related dementias.

Figure 6 illustrates the spatial distribution of AD-related mortality with hyperlipidemia across U.S. states in 2020. Figure 6A depicts the ASMR, with higher rates observed in the South-Central (e.g., Mississippi, Louisiana), Northeastern (e.g., Maine), and select Midwestern states, where rates frequently exceeded 10 per 100,000. In contrast, the West Coast (e.g., California, Washington) and Mountain states (e.g., Colorado) exhibited lower ASMRs. Figure 6B presents the AAPC by state during 1999–2020, revealing pronounced increases (AAPC >15%) in Southern (e.g., Texas, Alabama), Western (e.g., Arizona, Nevada), and Northeastern (e.g., New York, New Hampshire) states. Notably, some regions with currently low ASMRs (e.g., California, Colorado) displayed high AAPCs. Overall, the geographic distributions of ASMR and AAPC were not fully congruent (Supplementary Table 1).

## 4 Discussion

This study investigates the persistent upward trend in mortality related to ADRD comorbid with hyperlipidemia in the United States from 1999 to 2020, suggesting that this comorbidity is emerging as a significant public health burden. Although deaths from ADRD with hyperlipidemia currently constitute only a small proportion of all ADRD deaths, this proportion has risen steadily over time.

Comparisons with total ADRD mortality revealed distinct yet overlapping profiles. While total ADRD deaths also spiked after 2018—suggesting broad epidemiological drivers such as changes in reporting practices or pre-pandemic factors—the long-term rise in the ADRD-hyperlipidemia phenotype was substantially steeper. This divergence likely reflects improved clinical recognition of metabolic comorbidities in dementia patients, changes in coding practices for hyperlipidemia (E78), or a genuine epidemiological shift where metabolic dysregulation increasingly drives ADRD mortality. These findings highlight the need for integrated public health strategies that address both conditions simultaneously, especially in high-risk populations such as older adults, racial minorities, and those residing in regions with higher disease burden.

The observed epidemiological trends are supported by shared biological mechanisms. On one hand, hyperlipidemia accelerates AD-related neurodegenerative changes by promoting atherosclerosis, cerebral microangiopathy, and reduced cerebral blood flow. Conversely, neuroinflammatory processes in AD may disrupt metabolic pathways, thereby establishing a bidirectional pathogenic loop (20). Additionally, genetic susceptibility factors such as apolipoprotein E are closely associated with both hyperlipidemia and ADRD risk, which may further account for their frequent co-occurrence (21).

Gender disparities in mortality trends associated with ADRD and hyperlipidemia warrant close attention. Although women account for the majority of cases, men exhibit a more rapid increase in mortality, which may be partly attributed to their higher baseline risk for cardiovascular disease and delayed diagnosis and suboptimal management of ADRD (22, 23). Sociocultural factors may also contribute, as men are generally less likely to seek medical attention for early symptoms of cognitive decline, resulting in missed opportunities for timely diagnosis and intervention (24). This pronounced gender-specific trend suggests that future public health strategies should be optimized to enable sex-specific, precision-targeted prevention strategies. For instance, targeted early screening and aggressive control of cardiometabolic risk factors (e.g., hyperlipidemia) should be prioritized for middle-aged and older men, alongside heightened awareness of cognitive health.

From a racial perspective, this study reveals that Black populations exhibit both the highest mortality rates and the most rapid increases in mortality. This pattern aligns with previous research showing that age-adjusted mortality rates among Black individuals are significantly higher than those observed in White populations (25). Underlying this disparity are complex social determinants, including a high prevalence of metabolic syndrome (encompassing hyperlipidemia, hypertension, and diabetes), long-standing socioeconomic disadvantages, and structural barriers to equitable healthcare in African American communities (26). These factors collectively play a significant role in the onset, progression, and health outcomes of ADRD and hyperlipidemia. In contrast, although baseline mortality rates are lower among Asian Americans and Native Americans, their increasing mortality trends should not be overlooked. These findings underscore the need for tailored public health measures aimed at enhancing health literacy and expanding medical support for cognitive and metabolic health in these populations.

Geographically, mortality rates and temporal trends associated with ADRD and hyperlipidemia exhibit significant heterogeneity across U.S. states, reflecting regional differences in lifestyle factors (e.g., diet, physical activity), healthcare resource distribution, environmental exposures, and population demographics. For example, Southern and some Northeastern states exhibit higher ASMR and AAPC, which may be attributed to elevated rates of obesity, diabetes, and hyperlipidemia in these regions (27, 28). Conversely, states with lower ASMR but higher AAPC—such as California and Colorado—indicate a relatively low current burden but a high potential for escalation, underscoring the need for proactive public health interventions to address future challenges.

Urban–rural disparities further underscore systemic healthcare disparities. Nonmetropolitan areas not only exhibit higher ASMR but also show equal or greater increases in mortality compared to urban areas. Prior studies corroborate these findings, demonstrating that increases in mortality rates due to ADRD are more pronounced in rural regions than in all urban classifications, with the urban–rural gap widening over time (29, 30). These disparities may stem from higher aging rates, reduced capacity for chronic disease management, and diagnostic delays or suboptimal disease control caused by uneven healthcare resource distribution (31). Future intervention strategies should prioritize the strengthening of primary healthcare systems to support integrated prevention and management of ADRD and hyperlipidemia among older adults in rural areas.

Age-stratified analyses reveal that individuals aged 85 years and older account for the highest proportion of deaths and the steepest increases in mortality, highlighting their heightened vulnerability in the context of this comorbidity. Advanced age is often accompanied by cognitive decline, metabolic disorders, and complications due to polypharmacy, all of which substantially elevate mortality risk in individuals with both AD and hyperlipidemia (32, 33). Notably, although the majority of current deaths are concentrated among the oldest age group, long-term metabolic management during midlife plays a critical role in shaping future risk accumulation (34). Accordingly, it is imperative to develop integrated, life-course-based intervention strategies—emphasizing primary prevention in midlife and comprehensive disease management in older adulthood. Observed patterns may partly reflect nonbiological shifts—including updates to dementia diagnostic criteria, changes in cholesterol/lipid treatment guidelines (e.g., ATP III to ACC/AHA guidance), expanded EHR adoption with improved documentation/coding of co-morbidities, and increased public awareness leading to more formal diagnoses (35, 36). These factors could change ascertainment and coding of ADRD and hyperlipidemia on death certificates and thereby affect measured mortality trends (37). Our study is descriptive and does not attribute changes to these drivers; thus, late-period increases are interpreted cautiously.

From a policy perspective, this study offers valuable implications. First, integrated management strategies for AD and metabolic disorders should be reinforced by promoting collaboration across neurological and cardiometabolic care disciplines. Second, primary prevention and early screening efforts must be prioritized, particularly through integrated monitoring of cognitive and metabolic risk in high-risk groups such as men, racial minorities, residents of high-burden regions, and older adults. Third, addressing geographic and urban–rural disparities requires equitable resource allocation strategies to improve healthcare accessibility and equity. Additionally, future research should delve into the molecular mechanisms and interactive pathways that link ADRD and hyperlipidemia, aiming to elucidate causal pathways that can inform precision medicine approaches.

This study has several limitations. First, mortality data were derived from death certificates, which may be subject to diagnostic omissions, miscoding, or other coding inaccuracies. Deaths from ADRD with comorbid hyperlipidemia constituted a small yet growing proportion of total ADRD mortality. Between 1999 and 2020, this proportion increased tenfold, highlighting that the present analysis focuses on a specific comorbid subgroup rather than the entirety of ADRD-related deaths. Overall long-term changes in ADRD and other cardiometabolic diseases (such as hypertension and diabetes), alongside shifts in death certificate documentation and coding practices, may collectively influence the observed patterns—even though U.S. deaths for 1999 and beyond are classified using the ICD-10. Moreover, the lack of standardized coding guidelines for reporting hyperlipidemia as a contributing cause of ADRD-related deaths limits the reliability of comorbidity attribution. Second, as a population-level mortality analysis, individual-level behavioral data, medication histories, and detailed comorbidity profiles were not accessible, thereby constraining the ability to draw causal inferences. Third, this ecological trend analysis yields hypothesis-generating findings that require confirmation through prospective cohort studies and mechanistic research. Apparent late-period increases should also be interpreted in the context of broader mortality dynamics—most notably the COVID-19 pandemic in 2020, which disproportionately affected older adults. Future directions. Prospective cohorts plus record-linkage (Medicare/EHR–death certificates) are needed to validate individual-level associations and quantify misclassification; policy-aware causal/spatial analyses should test robustness and equity-relevant heterogeneity.

In summary, from 1999 to 2020, the United States experienced a persistent and significant rise in mortality associated with Alzheimer’s disease in combination with hyperlipidemia. This trend was particularly pronounced among older adults, men, African Americans, and individuals living in nonmetropolitan areas, accompanied by substantial regional variation. Future public health interventions must adopt targeted and precision-oriented strategies to address the dual burden of neurodegenerative and metabolic comorbidities through improved prevention, early detection, and coordinated care.

## Data Availability

The original contributions presented in the study are included in the article/Supplementary material, further inquiries can be directed to the corresponding authors.

## Footnotes

1
https://wonder.cdc.gov/mcd.html
