# Correction: Trends in mortality from Alzheimer's disease and related dementias with hyperlipidemia in the United States from 1999 to 2020—A CDC WONDER database study

**DOI:** 10.3389/fneur.2026.1856837

**Published:** 2026-05-06

**Authors:** Junwen Wang, Kaide Xia, Dianmei Yang, Jing Wu, Longfei Liu, Ying Huang, Qing Shan, Haiwang Zhang, Yiming Wang

**Affiliations:** 1 School of Clinical Medicine, Guizhou Medical University, Guiyang, China; 2 Department of Psychosomatic Medicine, The Second People's Hospital of Guiyang, Guiyang, China; 3 Guiyang Maternal and Child Health Care Hospital, Guiyang Children's Hospital, Guiyang, China; 4 Department of Endocrinology and Metabolism, Affiliated Hospital of Guizhou Medical University, Guiyang, China; 5 Department of Psychiatry, Affiliated Hospital of Guizhou Medical University, Guiyang, China; 6 Department of Neurosurgery, Guizhou Provincial People's Hospital, Guiyang, China

**Keywords:** Alzheimer's disease and related dementias, hyperlipidemia, ASMR, CDC WONDER, AAPC

In the published article, Figures 2–6 were presented in the wrong order. Consequently, the graphical content shown for these figures did not correspond to their captions and the related in-text figure citations in the Results section.

**Figure 2:**
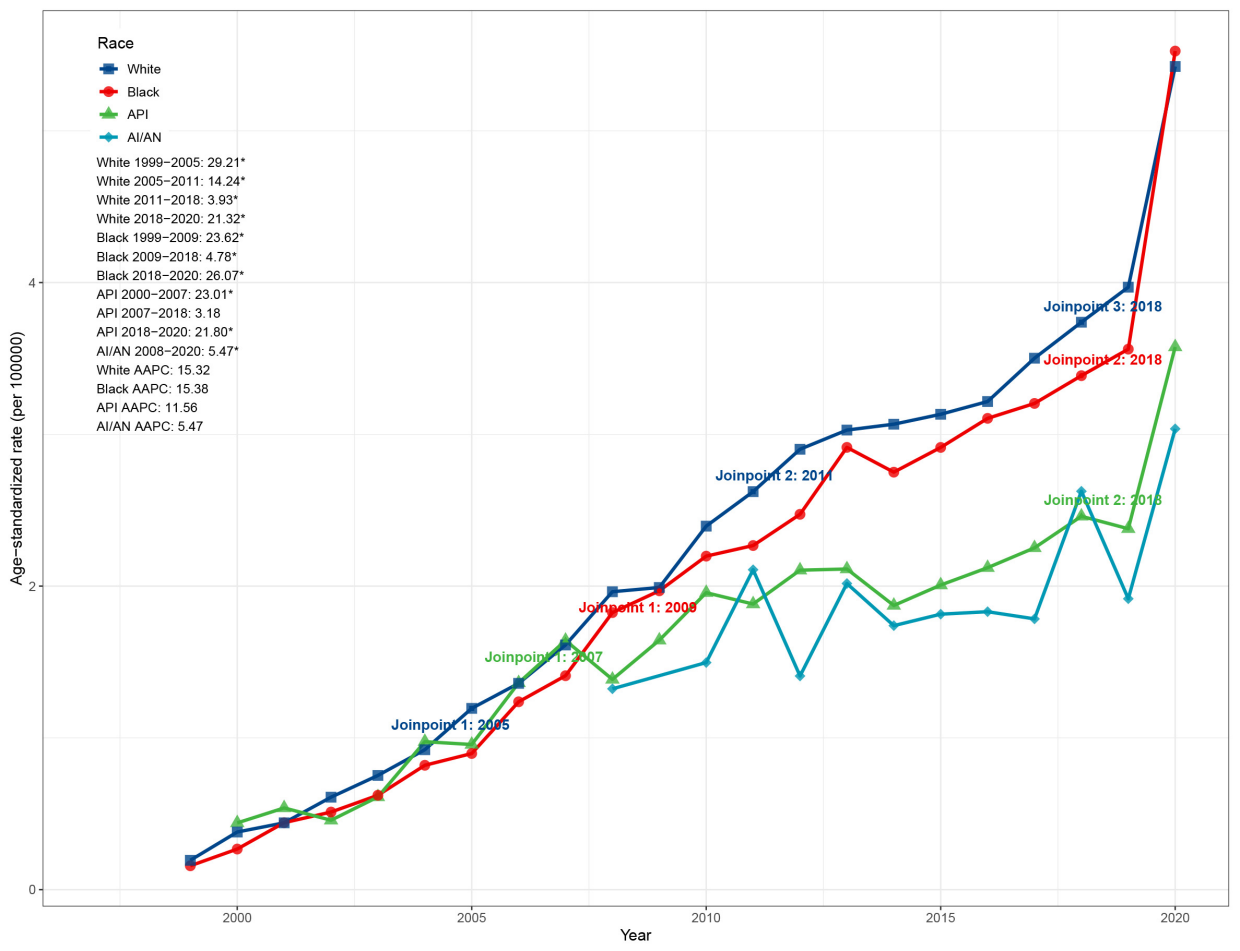
Race-specific trends in ASMR due to ADRD and hyperlipidemia in the United States, 1999–2020. ASMR, age-standardized mortality rate; ADRD, Alzheimer's disease and related dementias.

**Figure 3:**
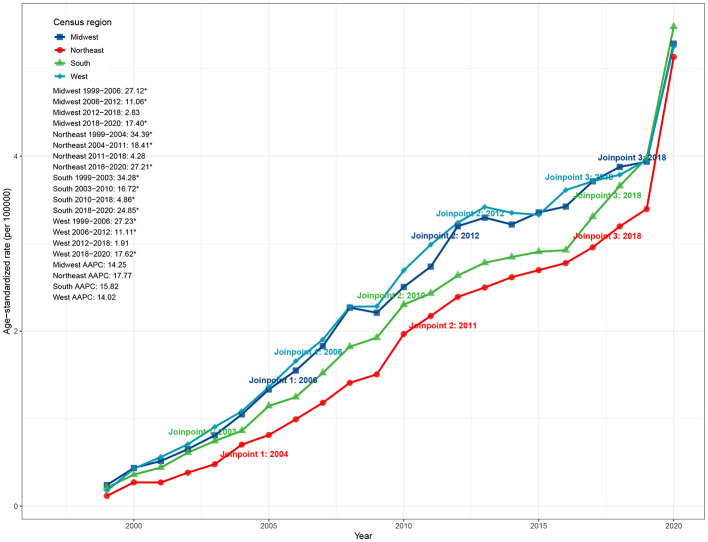
Census region trends in ASMR due to ADRD and hyperlipidemia in the United States, 1999–2020. ASMR, age-standardized mortality rate; ADRD, Alzheimer's disease and related dementias.

**Figure 4:**
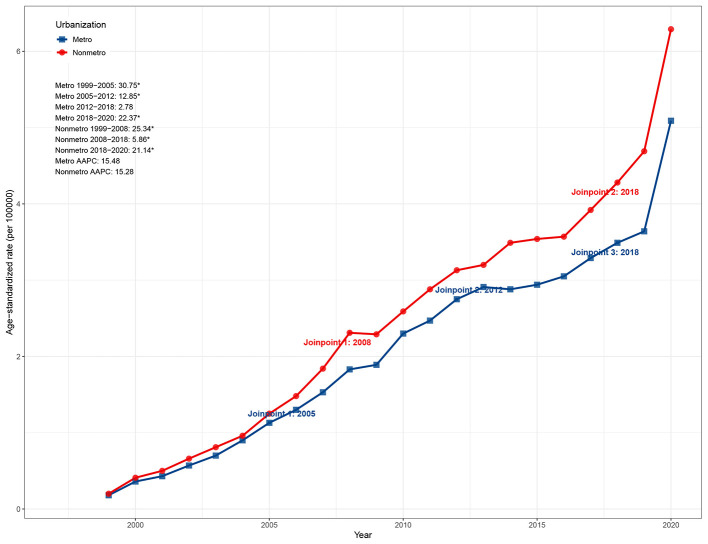
Urban–rural disparities in ASMR due to ADRD and hyperlipidemia in the United States, 1999–2020. ASMR, age-standardized mortality rate; ADRD, Alzheimer's disease and related dementias.

**Figure 5:**
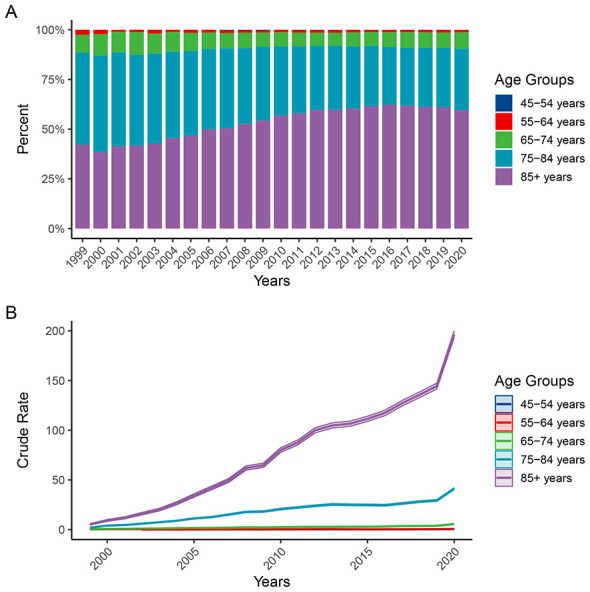
Trends in the proportion **(A)** and crude mortality rate **(B)** of ADRD-related deaths with hyperlipidemia across age groups in the United States, 1999–2020. ADRD, Alzheimer's disease and related dementias.

**Figure 6:**
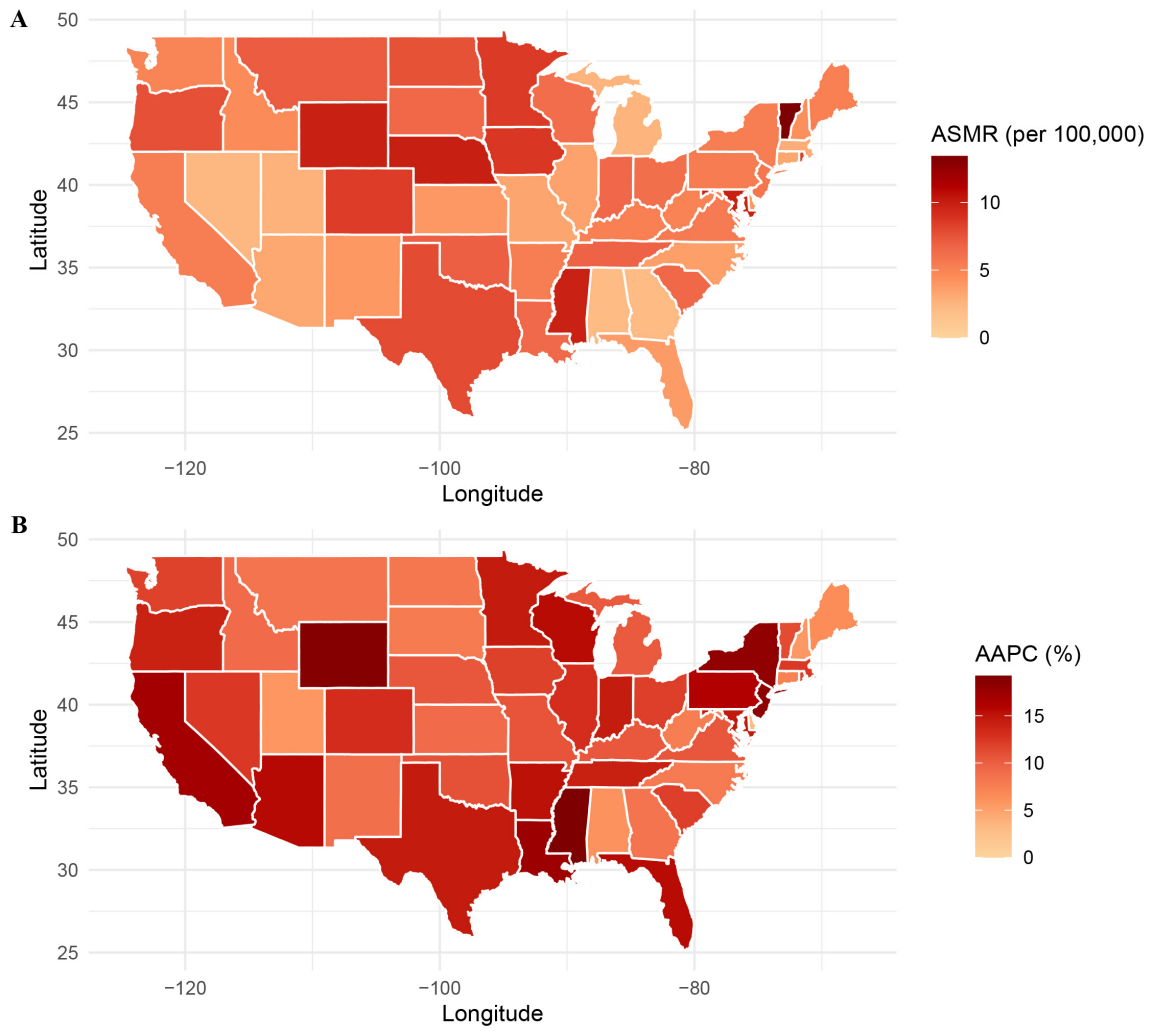
Geographic region–specific trends in ASMR **(A)** and AAPC **(B)** due to ADRD and hyperlipidemia in the United States, 1999–2020. ASMR, age-standardized mortality rate; ADRD, Alzheimer's disease and related dementias.

This error affected the presentation order of the figures only. The figure captions, figure numbers, and the in-text descriptions in the Results section remain unchanged.

The order of Figures 2–6 has now been corrected.

The original version of this article has been updated.

