# Supplementary material for: Trends in mortality from Alzheimer’s disease and related dementias with hyperlipidemia in the United States from 1999 to 2020—a CDC WONDER database study
Source: Front Neurol. 2025 Dec 17;16:1705607. doi: 10.3389/fneur.2025.1705607 (PMC12753401; doi:10.3389/fneur.2025.1705607)
Supplement: Supplementary file 1 [file Supplementary_file_1.docx]

**Supplementary material**

**Trends in Mortality from Alzheimer’s Disease and Related Dementias with Hyperlipidemia in the United States from 1999 to 2020 – A CDC WONDER Database Study**

Junwen Wang^1, 4†^, Kaide Xia^2†^, Dianmei Yang^3^, Jing Wu^1^, Longfei Liu^1^, Ying Huang^4^, Qing Shan^5^, Haiwang Zhang^6*†^, Yiming Wang^1,5*†^

^1^ School of Clinical Medicine, Guizhou Medical University, Guiyang, China.

^2^ Guiyang Maternal and Child Health Care Hospital, Guiyang Children's Hospital, Guiyang, China.

^3^ Department of Endocrinology and Metabolism, Affiliated Hospital of Guizhou Medical University, Guiyang, China.

^4^ Department of Psychosomatic Medicine, The Second People’s Hospital of Guiyang, Guiyang, China.

^5^ Department of Psychiatry, Affiliated Hospital of Guizhou Medical University, Guiyang, China.

^6^ Department of Neurosurgery, Guizhou Provincial People's Hospital, Guiyang, China.

**Content**

[**Supplementary Figures** 2](#_Toc214653992)

[**Supplementary Figure 1. Trends in the proportion of ADRD deaths co-listing hyperlipidemia in the United States, 1999–2020.** 2](#_Toc214653993)

[**Supplementary Figure 3. rends in ASMRs due to overall ADRD by sex in the United States, 1999–2020.** 3](#_Toc214653994)

[**Supplementary Figure 4. Trends in ASMRs due to overall ADRD by race in the United States, 1999–2020.** 4](#_Toc214653995)

[**Supplementary Figure 5. Trends in ASMRs due to overall ADRD by census regions in the United States, 1999–2020.** 5](#_Toc214653996)

[**Supplementary Figure 6. Trends in ASMRs due to overall ADRD by urbanizaition in the United States, 1999–2020.** 6](#_Toc214653997)

[**Supplementary Tables** 7](#_Toc214653998)

[**Supplementary Table 1. State-level trends in ASMRs due to ADRD with hyperlipidemia in the United States, 1999–2020.** 7](#_Toc214653999)

# **Supplementary Figures**


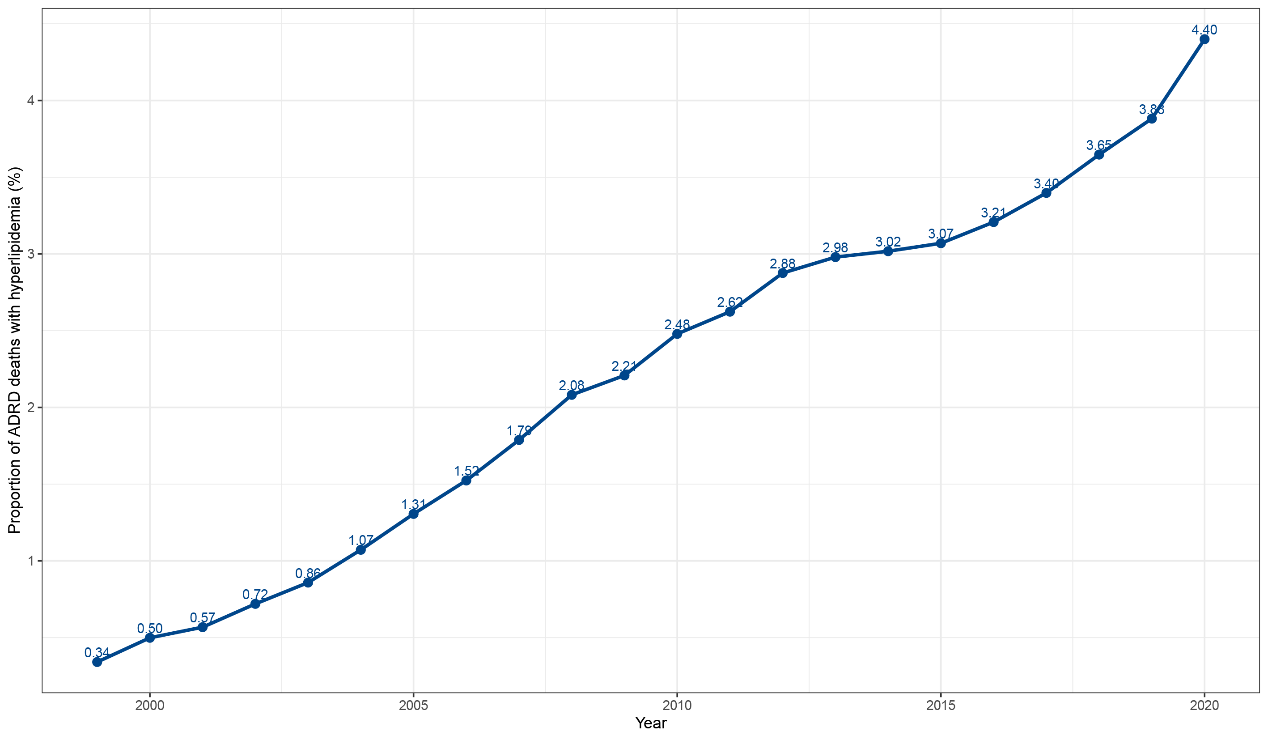


**Supplementary Figure 1. Trends in the proportion of ADRD deaths co-listing hyperlipidemia in the United States, 1999–2020.** ADRD, alzheimer’s disease and related dementias.


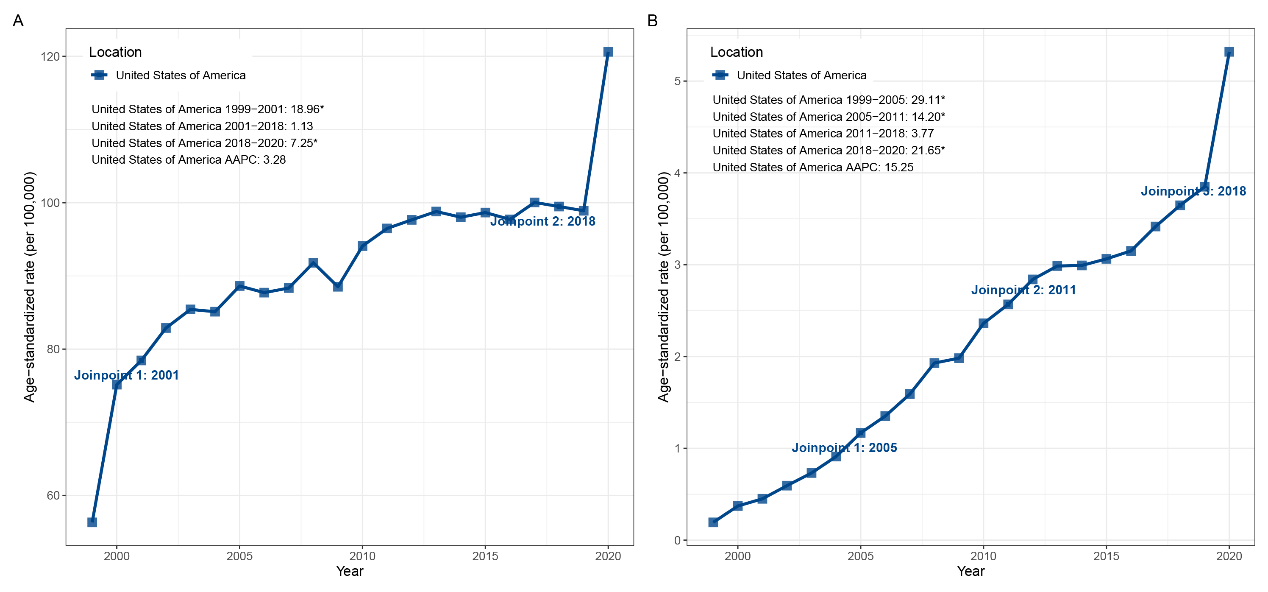
**Supplementary Figure 2. Trends in ASMRs due to overall ADRD (A) and ADRD with hyperlipidemia (B) in the United States, 1999–2020.** ASMR, age-standardized mortality rate; ADRD, alzheimer’s disease and related dementias.


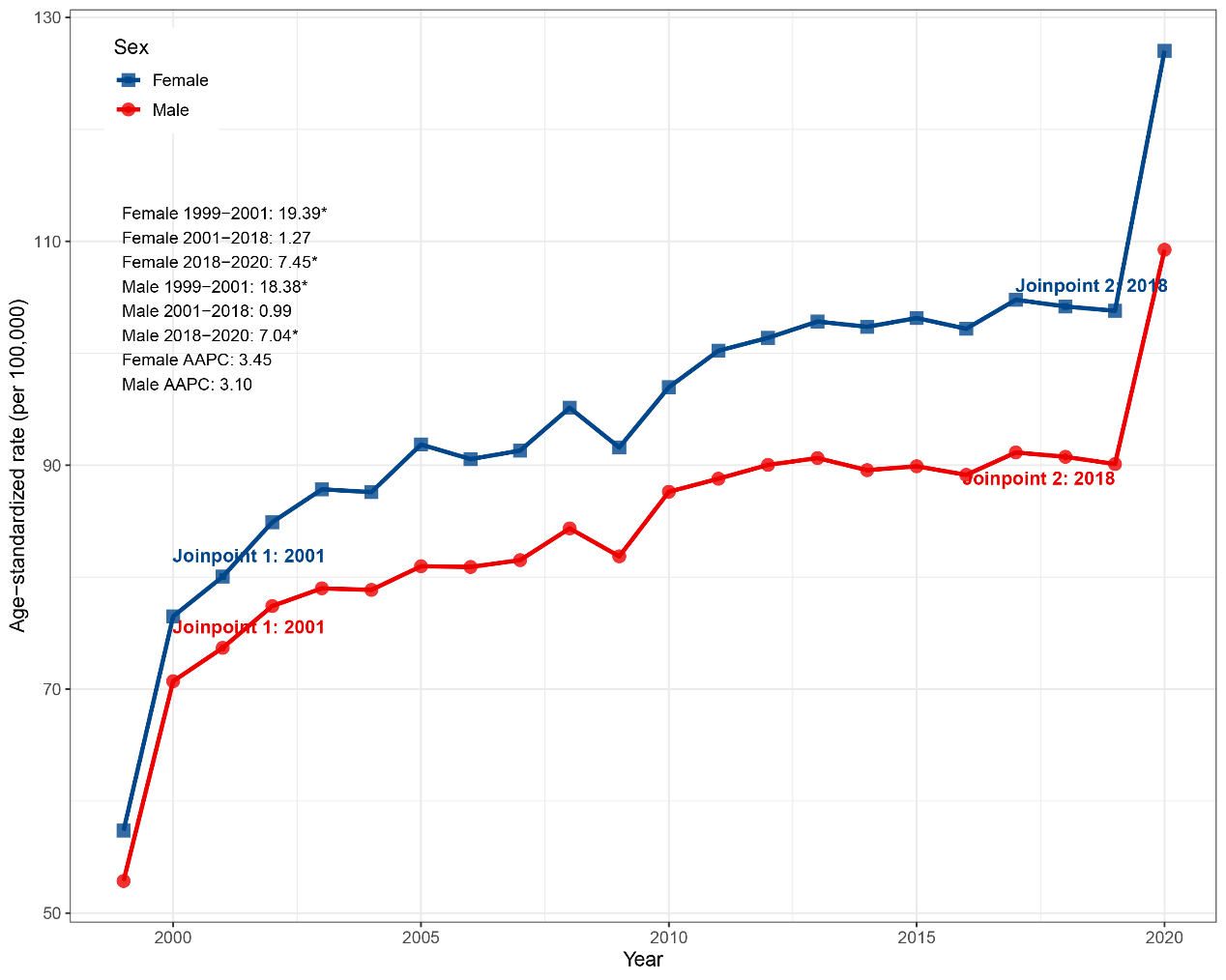


**Supplementary Figure 3. rends in ASMRs due to overall ADRD by sex in the United States, 1999–2020.** ASMR, age-standardized mortality rate; ADRD, alzheimer’s disease and related dementias.


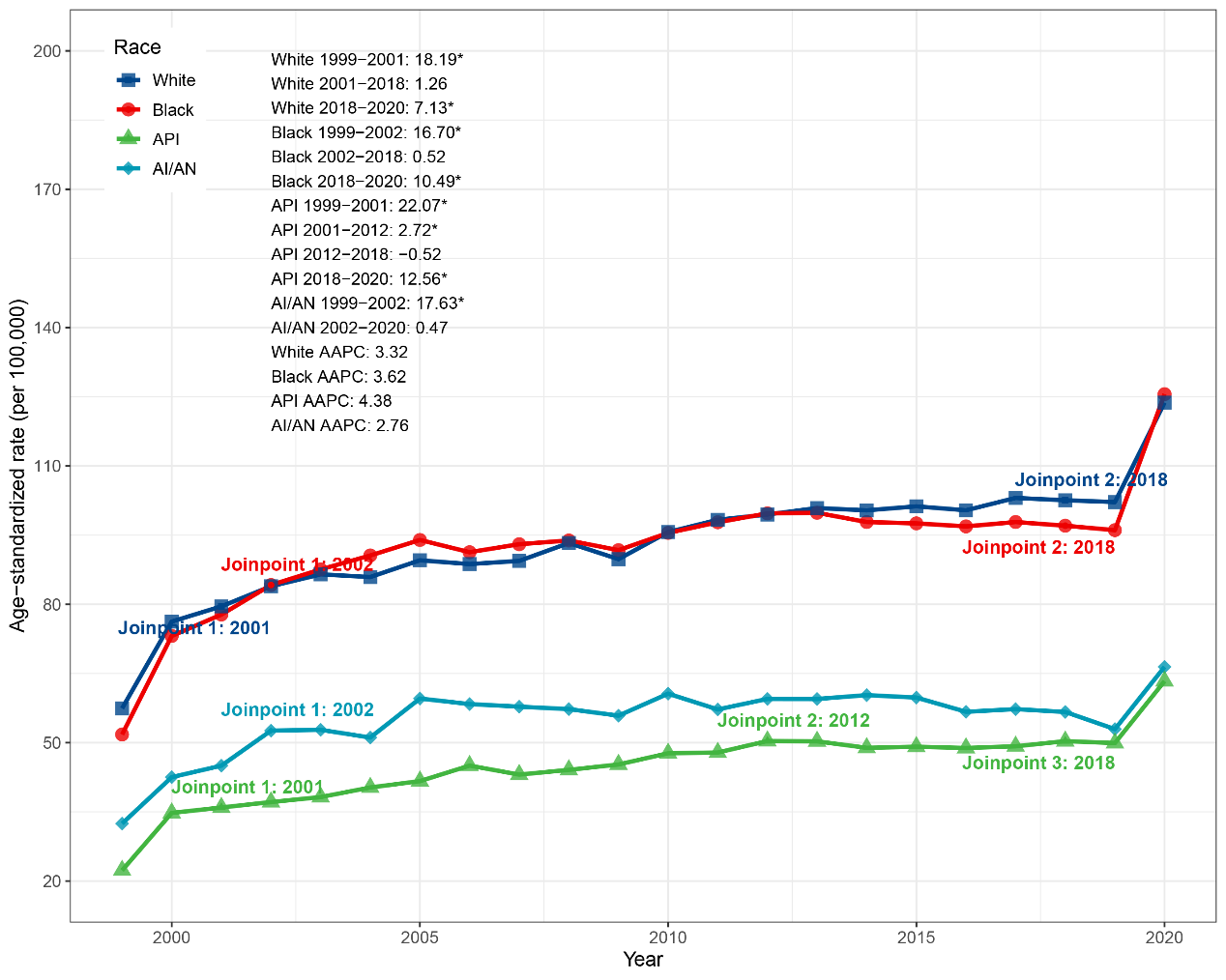


**Supplementary Figure 4. Trends in ASMRs due to overall ADRD by race in the United States, 1999–2020.** ASMR, age-standardized mortality rate; ADRD, alzheimer’s disease and related dementias.


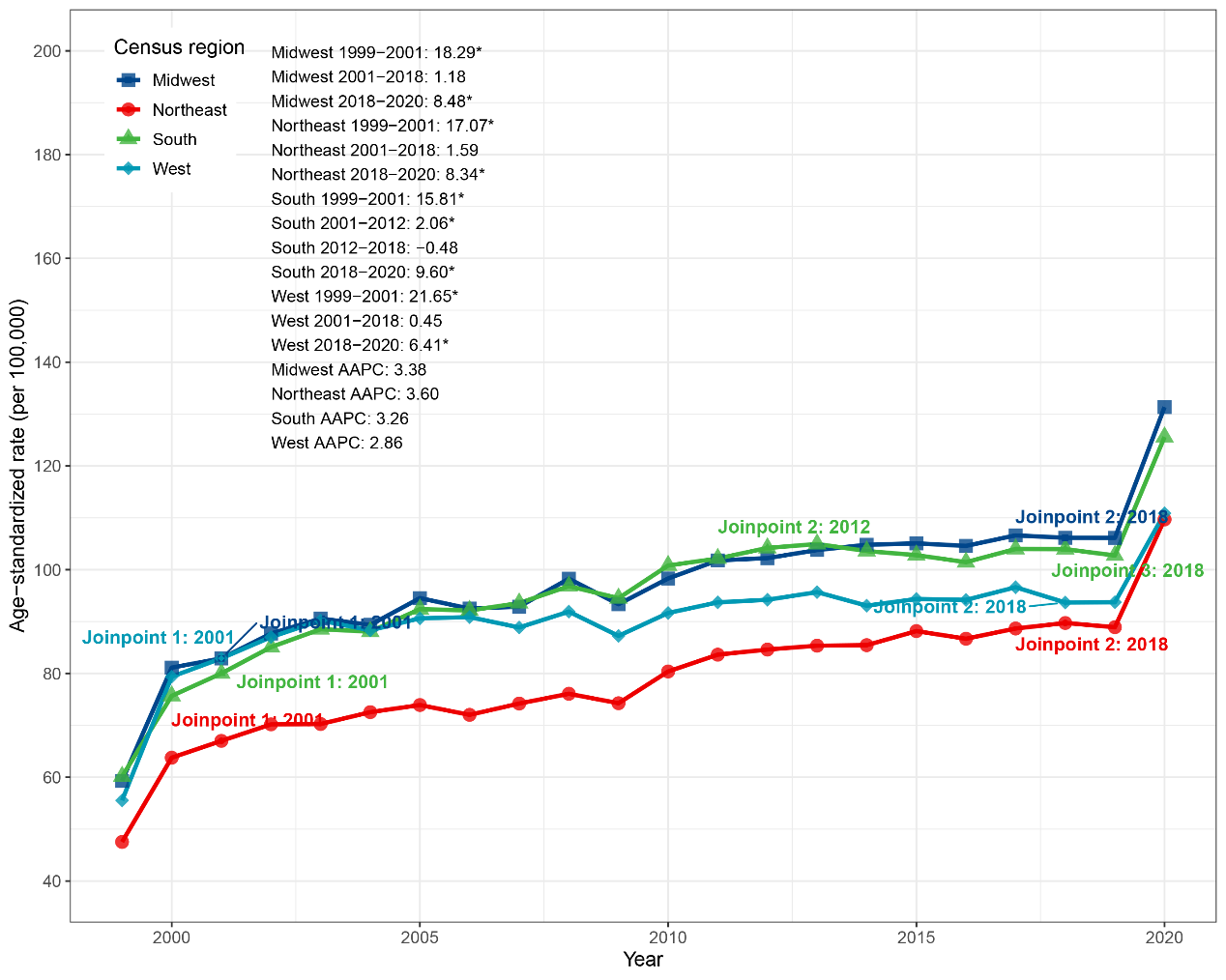


**Supplementary Figure 5. Trends in ASMRs due to overall ADRD by census regions in the United States, 1999–2020.** ASMR, age-standardized mortality rate; ADRD, alzheimer’s disease and related dementias.


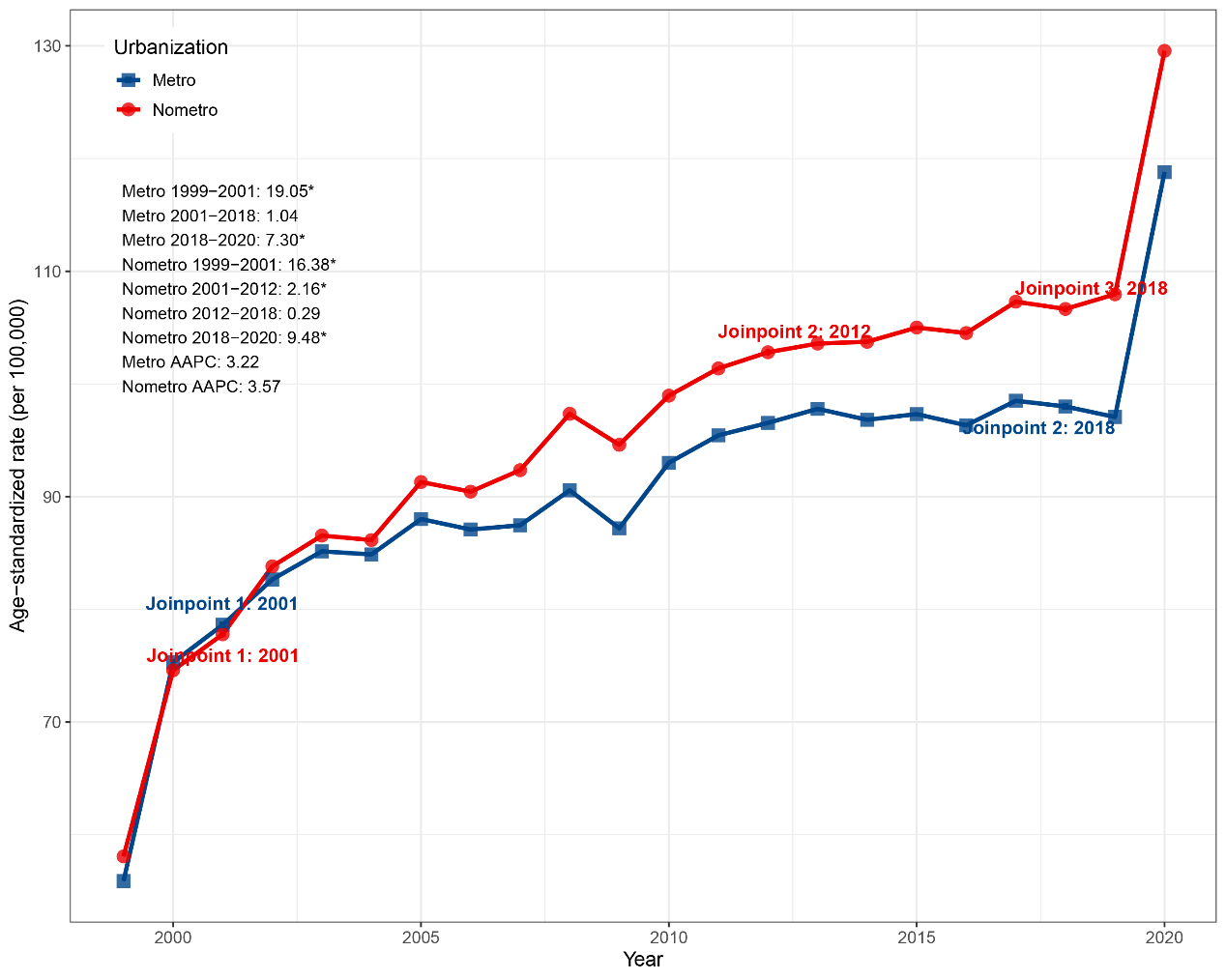


**Supplementary Figure 6. Trends in ASMRs due to overall ADRD by urbanizaition in the United States, 1999–2020.** ASMR, age-standardized mortality rate; ADRD, alzheimer’s disease and related dementias.

# **Supplementary Tables**

| **Supplementary Table 1. State-level trends in ASMRs due to ADRD with hyperlipidemia in the United States, 1999–2020.** | | | | | | | | |
| --- | --- | --- | --- | --- | --- | --- | --- | --- |
| **State** | **Initial** | | |  | **2020** | |  | **AAPC** |
|  | **Year** | **Number** | **ASMR** |  | **Number** | **ASMR** |  |  |
| California | 1999 | 43 | 0.16 (0.11, 0.21) |  | 2444 | 5.32 (5.10, 5.53) |  | 16.9(15.02 to 20.62) |
| Florida | 1999 | 26 | 0.12 (0.08, 0.18) |  | 1398 | 3.79 (3.59, 3.99) |  | 15.55(14.2 to 18.79) |
| Illinois | 1999 | 22 | 0.19 (0.12, 0.28) |  | 549 | 3.46 (3.16, 3.75) |  | 13.06(10.77 to 17.25) |
| Maryland | 1999 | 20 | 0.44 (0.27, 0.68) |  | 727 | 9.82 (9.11, 10.54) |  | 14.64(13.31 to 17.29) |
| Michigan | 1999 | 24 | 0.26 (0.17, 0.38) |  | 332 | 2.52 (2.25, 2.79) |  | 10.38(8.89 to 12.6) |
| Ohio | 1999 | 37 | 0.31 (0.22, 0.43) |  | 963 | 6.17 (5.78, 6.56) |  | 12.01(10.62 to 14.96) |
| Pennsylvania | 1999 | 22 | 0.14 (0.09, 0.22) |  | 1064 | 5.46 (5.12, 5.79) |  | 16.08(14.66 to 20.47) |
| Tennessee | 1999 | 20 | 0.38 (0.23, 0.58) |  | 556 | 6.83 (6.26, 7.40) |  | 14.16(13.18 to 15.84) |
| Texas | 1999 | 48 | 0.32 (0.23, 0.42) |  | 2107 | 7.90 (7.56, 8.24) |  | 14.6(13.97 to 15.7) |
| Indiana | 2000 | 25 | 0.42 (0.27, 0.63) |  | 540 | 6.62 (6.06, 7.18) |  | 14.48(12.95 to 17.06) |
| Minnesota | 2000 | 22 | 0.43 (0.27, 0.66) |  | 626 | 8.60 (7.92, 9.28) |  | 14.57(13.21 to 17.57) |
| Missouri | 2000 | 20 | 0.33 (0.20, 0.51) |  | 273 | 3.31 (2.91, 3.70) |  | 10.78(9.27 to 13.86) |
| New York | 2000 | 35 | 0.19 (0.13, 0.26) |  | 1442 | 5.31 (5.04, 5.59) |  | 18.09(16.66 to 21.7) |
| North Carolina | 2000 | 58 | 0.79 (0.60, 1.03) |  | 455 | 3.62 (3.29, 3.96) |  | 7.97(7.02 to 9.25) |
| Oklahoma | 2000 | 23 | 0.63 (0.40, 0.95) |  | 334 | 7.00 (6.24, 7.75) |  | 10.93(9.48 to 13.43) |
| Oregon | 2000 | 20 | 0.55 (0.34, 0.85) |  | 419 | 7.68 (6.94, 8.42) |  | 14.07(12.46 to 16.5) |
| Washington | 2000 | 33 | 0.62 (0.43, 0.87) |  | 435 | 5.00 (4.53, 5.48) |  | 11.87(10.8 to 14.83) |
| Wisconsin | 2000 | 21 | 0.37 (0.23, 0.56) |  | 488 | 6.28 (5.72, 6.84) |  | 15.54(13.51 to 19.7) |
| Colorado | 2002 | 20 | 0.60 (0.37, 0.92) |  | 501 | 8.50 (7.76, 9.25) |  | 13.18(10.92 to 16.09) |
| Georgia | 2002 | 24 | 0.39 (0.25, 0.58) |  | 236 | 2.23 (1.95, 2.52) |  | 8.46(6.72 to 11.68) |
| Iowa | 2002 | 27 | 0.71 (0.47, 1.04) |  | 395 | 8.75 (7.88, 9.62) |  | 11.92(10.23 to 14.7) |
| Massachusetts | 2002 | 22 | 0.29 (0.18, 0.45) |  | 276 | 2.92 (2.57, 3.26) |  | 12.36(11.14 to 14.6) |
| New Jersey | 2002 | 20 | 0.22 (0.14, 0.34) |  | 689 | 5.80 (5.36, 6.23) |  | 18.14(16 to 23.02) |
| South Carolina | 2002 | 32 | 0.86 (0.59, 1.22) |  | 423 | 6.58 (5.95, 7.21) |  | 12.01(10.71 to 14.37) |
| Virginia | 2002 | 52 | 0.85 (0.63, 1.11) |  | 560 | 5.57 (5.11, 6.04) |  | 10.58(8.98 to 14.79) |
| Alabama | 2003 | 28 | 0.62 (0.41, 0.89) |  | 136 | 2.24 (1.86, 2.61) |  | 6.06(4.51 to 8.45) |
| Connecticut | 2003 | 22 | 0.53 (0.33, 0.81) |  | 171 | 3.25 (2.76, 3.74) |  | 7.32(5.84 to 9.48) |
| Hawaii | 2003 | 23 | 1.69 (1.07, 2.53) |  | 154 | 6.17 (5.18, 7.16) |  | 6.47(4.3 to 11.57) |
| Kansas | 2003 | 27 | 0.88 (0.58, 1.28) |  | 152 | 3.94 (3.30, 4.57) |  | 9.22(7.52 to 11.79) |
| Kentucky | 2003 | 35 | 0.89 (0.62, 1.24) |  | 281 | 5.21 (4.60, 5.82) |  | 10.49(9.22 to 13.79) |
| West Virginia | 2003 | 25 | 1.17 (0.76, 1.73) |  | 133 | 5.07 (4.21, 5.94) |  | 7.68(5.91 to 10.8) |
| Arizona | 2004 | 23 | 0.42 (0.27, 0.64) |  | 317 | 3.19 (2.84, 3.54) |  | 15.62(12.11 to 23.88) |
| Maine | 2004 | 23 | 1.50 (0.95, 2.25) |  | 108 | 5.21 (4.22, 6.20) |  | 6.42(3.37 to 9.18) |
| North Dakota | 2004 | 21 | 2.51 (1.53, 3.87) |  | 81 | 7.61 (6.02, 9.50) |  | 8.19(6.72 to 11.62) |
| Rhode Island | 2004 | 20 | 1.52 (0.93, 2.35) |  | 136 | 8.46 (7.02, 9.89) |  | 11.87(8.27 to 17.67) |
| Arkansas | 2005 | 27 | 0.90 (0.59, 1.31) |  | 209 | 5.36 (4.63, 6.09) |  | 15.24(13.29 to 18.6) |
| Nebraska | 2005 | 48 | 2.31 (1.70, 3.08) |  | 247 | 9.92 (8.67, 11.17) |  | 10.56(6.57 to 16.73) |
| New Hampshire | 2006 | 31 | 2.34 (1.59, 3.33) |  | 83 | 4.34 (3.45, 5.39) |  | 5.67(2.94 to 9.26) |
| Idaho | 2007 | 22 | 1.55 (0.97, 2.35) |  | 95 | 4.61 (3.73, 5.64) |  | 9.17(7.28 to 11.99) |
| Montana | 2007 | 21 | 1.86 (1.15, 2.84) |  | 105 | 7.15 (5.78, 8.52) |  | 8.47(4.65 to 13.68) |
| New Mexico | 2007 | 24 | 1.27 (0.82, 1.89) |  | 108 | 3.98 (3.23, 4.73) |  | 8.88(5.97 to 11.79) |
| Vermont | 2007 | 25 | 3.63 (2.35, 5.36) |  | 122 | 13.43 (11.04, 15.82) |  | 11.28(7.48 to 20.09) |
| Louisiana | 2008 | 37 | 0.91 (0.64, 1.25) |  | 346 | 6.48 (5.80, 7.17) |  | 17.37(14.9 to 21.43) |
| Mississippi | 2008 | 35 | 1.24 (0.87, 1.73) |  | 344 | 9.85 (8.80, 10.89) |  | 19.23(17.59 to 21.81) |
| South Dakota | 2008 | 27 | 2.51 (1.64, 3.68) |  | 81 | 6.55 (5.19, 8.16) |  | 7.89(5.59 to 10.84) |
| Delaware | 2009 | 25 | 2.58 (1.67, 3.80) |  | 53 | 3.91 (2.93, 5.11) |  | 2.98(0.38 to 6.11) |
| Utah | 2010 | 29 | 1.50 (1.00, 2.15) |  | 71 | 2.73 (2.13, 3.45) |  | 5.8(3.21 to 9.82) |
| Nevada | 2011 | 24 | 1.00 (0.64, 1.51) |  | 81 | 2.40 (1.90, 2.99) |  | 12.4(6.14 to 22.3) |
| District of Columbia | 2013 | 22 | 3.33 (2.06, 5.09) |  | 41 | 5.59 (3.99, 7.61) |  | 7.26(-1.7 to 20.19) |
| Wyoming | 2016 | 28 | 4.59 (3.05, 6.64) |  | 69 | 9.85 (7.65, 12.49) |  | 18.85(1.16 to 45.51) |
| Note: Because of data unavailability in certain states during the early period, the analysis used the earliest year with sufficient data available for each state. | | | | | | | | |
